# Clinicohematologic and molecular response of essential thrombocythemia patients treated with pegylated interferon-α: a multi-center study of the German Study Group-Myeloproliferative Neoplasms (GSG-MPN)

**DOI:** 10.1038/s41375-023-01837-9

**Published:** 2023-02-24

**Authors:** Frank Stegelmann, Lino L. Teichmann, Florian H. Heidel, Carl C. Crodel, Thomas Ernst, Sebastian Kreil, Andreas Reiter, Sara Otten, Stefanie Schauer, Ruth-Miriam Körber, Kim Kricheldorf, Susanne Isfort, Hartmut Döhner, Tim H. Brümmendorf, Martin Griesshammer, Konstanze Döhner, Steffen Koschmieder

**Affiliations:** 1 Department of Internal Medicine III, University Hospital of Ulm, Ulm, Germany; 2 Department of Medicine III, University Hospital Bonn, Bonn, Germany; 3 Center for Integrated Oncology Aachen Bonn Cologne Düsseldorf (CIO ABCD), Aachen/Bonn, Germany; 4 Innere Medizin C, Hämatologie und Onkologie, Universitätsmedizin Greifswald, Greifswald, Germany; 5 Innere Medizin 2, Hämatologie und Onkologie, Universitätsklinikum Jena, Jena, Germany; 6 Department of Hematology and Oncology, University Hospital Mannheim, Mannheim, Germany; 7 Department of Hematology, Oncology, Hemostaseology, and Stem Cell Transplantation, Faculty of Medicine, RWTH Aachen University, Aachen, Germany; 8 University Clinic for Hematology, Oncology, Haemostaseology and Palliative Care, Johannes Wesling Medical Center Minden, University of Bochum, Bochum, Germany

**Keywords:** Myeloproliferative disease, Cancer immunotherapy

## To the Editor:

Although the prognosis of essential thrombocythemia (ET) is generally favorable, the risk of disease progression to secondary myelofibrosis (SMF) or acute myeloid leukemia (AML) increases over time, which makes their prevention particularly important for patients diagnosed at a younger age [1, 2].

Treatment with hydroxyurea (HU) or anagrelide (ANA) aims at normalizing the platelet (PLT) count and decreasing the risk of vascular complications. However, long-term intake of HU is associated with multiple side effects leading to discontinuation in half of the patients [3]. Furthermore, HU and ANA do not modify the natural course of ET, whereas interferon-α (IFN) may also prevent disease progression to SMF, as recently shown for polycythemia vera (PV) [4, 5]. This notion is supported by the observation of molecular responses (MR) in a subset of ET patients (partial MR [PMR], i.e., ≥50% allele burden reduction, in 15–57% and complete MR [CMR] in up to 33% of patients) [6–9].

Pegylated formulations of IFN (pegIFN) reduce treatment-related adverse events and allow for the extension of the application intervals. However, the accessibility to pegIFN is currently limited to PV patients due to a lack of approval in ET.

In a recent meta-analysis of 30 ET studies conducted between 1990 and 2014, data on the use of IFN were promising, including complete hematologic response rates of 59% and an annual discontinuation rate due to adverse events of 9% [9]. Although this analysis included a total number of 730 ET patients, there are critical limitations: (i) the majority of patients (490/730, 67%) were treated with non-pegylated IFN, (ii) the median number of patients per study was low (*n* = 20), and (iii) the time of follow-up was limited (median 24 months for pegIFN studies, range 9–83).

To investigate the clinical benefits of pegIFN in ET, we here report the results from a retrospective analysis of a large cohort of 127 ET patients treated in routine clinical practice at seven academic centers. All patients provided written informed consent to the German Study Group-Myeloproliferative Neoplasms (GSG-MPN) registry (NCT03125707). ET was diagnosed after bone marrow biopsy in all cases based on the latest WHO classification at that time.

The median age of patients at ET diagnosis was 37.0 years (range, 8.2–77.4); 67% were female and 33% were male (Table 1). Median baseline PLT count and median white blood cell (WBC) count at the start of pegIFN was 780/nL (124–2776) and 8.2/nL (3.0–17.3), respectively. The presence of immature white or red precursor cells in the peripheral blood was excluded in all patients. Furthermore, 51% of the patients were *JAK2* V617F, 32% *CALR*, and 5% *MPL* mutated. According to the International Prognostic Score of thrombosis in ET (IPSET-thrombosis), 37% were low-, 31% intermediate-, and 30% high-risk (2% unknown) [10]. Splenomegaly, as assessed by clinical palpation or ultrasound, was present in 29% of the patients (median diameter by ultrasound 11.05 cm).

**Table 1:** Main characteristics of 127 patients treated with pegylated interferon-α (pegIFN).

Number of unique patients	127
Total number of pegIFN LOT	161
Sex, *n* (%)	
Female	85 (67%)
Male	42 (33%)
Median age at diagnosis, years (min, max)	37.0 (8.2, 77.4)
IPSET-thrombosis score, %	
Low	37
Intermediate	31
High	30
Unknown	2
Driver mutation, %	
* JAK2* V617F	51
* CALR* Ex9	32
* JAK2* V617F and *CALR* Ex9	2
* MPL* W515	5
Triple negative	10
Splenomegaly, %	
Yes	29
No	68
Unknown	3
Smoking during pegIFN treatment, %	
Yes	11
No	72
Unknown	17
Median WBC, G/l (min, max)^a^	8.2 (3.0, 17.3)
Median PLT count, G/l (min, max)^a^	780 (124, 2776)
Median time until pegIFN start, years (min, max)	1.9 (0.0, 32.7)
Median total pegIFN treatment duration per patient, years (min, max)	2.3 (0.1, 18.1)
Reason for pegIFN start, %	
Platelet count	39
Prior thrombosis	31
Symptoms	9
Age >60 years	7
Pregnancy	7
Unknown	7
Prior cytoreductive therapies, %	
Hydroxyurea only	20
Anagrelide only	9
Hydroxyurea and anagrelide	11
Busulfan	1
None	59
Response according to Barosi 2009 criteria, %	
Complete response	54
Partial response	35
No response	9
Unknown	2
Vascular events per patient-year, %	
Arterial	0.64
Venous	1.07
Progression to SMF per patient-year, %	0.21
Leukemic transformation per patient-year, %	0.00
Adverse events, % LOT	
Flu-like symptoms	41
Abnormal liver values	16
Depression	14

^a^Data available from 110 patients.

According to the high-risk definition of the European LeukemiaNet (ELN) cytoreductive treatment was indicated in patients at age >60 years, with a history of thrombosis or major bleeding, and/or PLT count >1500/nL [11]. Median time from ET diagnosis to start of pegIFN treatment was 1.9 years (0.0–37.2). Notably, 59% of the patients received pegIFN as first-line treatment, 20% were pre-treated with HU, 9% with ANA, and 11% with both; one patient had received busulfan prior to pegIFN. Main reasons for starting pegIFN treatment in our study were extreme thrombocytosis in 39%, prior thrombosis in 31%, persistent ET-associated symptoms (e.g., microcirculatory disturbances) in 9%, age >60 years in 7%, and pregnancy in 7% (change from other cytoreductive treatment) of cases.

**Fig. 1:**
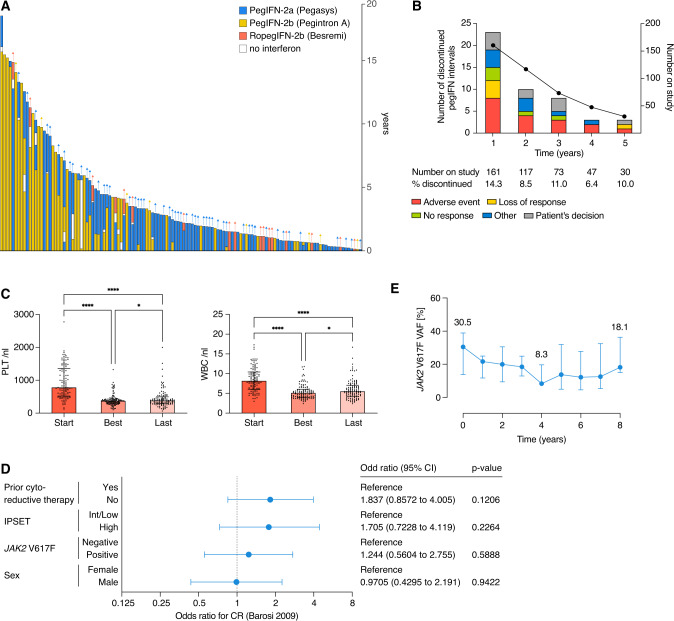
Time on pegylated interferon-α (pegIFN), treatment discontinuations, blood counts, correlations of parameters with achievement of clinicohematologic response in pegIFN-treated essential thrombocythemia (ET) patients, and course of the *JAK2* V617F variant allele frequency (VAF) over time. **A** Swimmer plot for time on pegIFN for 127 ET patients. **B** Proportion of ET patients who discontinued pegIFN by year and reasons for treatment discontinuation. Market withdrawal of pegIFN-2b was excluded as reason for discontinuation in this illustration. **C** Platelet (PLT) and white blood cell (WBC) counts at start, best response, and last follow-up in 110 patients with ET treated with pegIFN. Data are represented as median with interquartile range. Statistically significant differences were determined by Friedman’s test followed by Dunn’s multiple comparison test (*p < 0.05, ****p < 0.0001). **D** Effect of prior cytoreductive therapy, IPSET-thrombosis category, *JAK2* V617F mutation status and sex on achieving a complete clinicohematologic response with pegIFN. Multivariate logistic regression analysis was used to determine odds ratios and 95% confidence intervals. **E** *JAK2* V617F VAF before and until 8 years after pegIFN start in 14 patients for which samples were available measured by ddPCR (sensitivity of 10^−3^). Data are represented as median with interquartile range.

Since 31 patients (24%) had ≥2 lines of treatment (LOT) with pegIFN, overall 161 LOT were recorded in 127 patients (Fig. 1A). PegIFN-2a was used in most LOT (54%), followed by pegIFN-2b (35%) and ropegIFN-2b (11%). The median total pegIFN treatment duration per patient was 2.3 years (0.1–18.1). A considerable proportion of patients received pegIFN over an extended period of time: 30/127 patients (24%) were treated for at least 5 years and 10/127 (8%) for more than 10 years. At the last follow-up, 60% of patients were still on pegIFN, while 40% dropped out. The discontinuation rate per year was highest in the first year of pegIFN treatment (14.3%) and ranged between 6.4% and 11% in the years 2–5 (Fig. 1B). Adverse events (16%), market withdrawal of pegIFN-2b (15%) and patient’s decision (7%) were the three most frequent reasons for discontinuing a LOT, followed by no response (4%) or loss of response (3%).

According to ELN response criteria, 89% of patients achieved clinicohematologic response at any time point during pegIFN treatment, which was complete in 54% and partial in 35% of patients (9% no response, 2% unknown) [12]. The median PLT and WBC count at the last follow-up (380/nL and 5.5/nL) and at the best response (369/nL and 5.1/nL) were considerably lower compared to assessment before pegIFN administration (780/nL and 8.2/nL) (Fig. 1C). Data on pegIFN dosing as well as on the time point of best response were not available.

Notably, just one disease progression to SMF and no transformation to sAML occurred in 469 pegIFN treatment years. Major vascular complications were also rare, with three arterial and five venous events resulting in an incidence of 0.6% and 1.1% per patient and year, respectively. Of interest, all arterial events were thromboembolic myocardial infarctions, whereas 4/5 venous complications were abdominal vein thromboses (one pulmonary embolism). Flu-like symptoms occurred in 41%, elevated liver function tests in 16% and depression in 14% of LOT (grading and time points of events not assessed).

Furthermore, we calculated odds ratios for the achievement of complete clinicohematologic response with respect to prior cytoreductive therapy, IPSET-thrombosis risk category, *JAK2* mutation status, and sex (Fig. 1D): Although significance was not reached, these analyses show a favorable trend for patients without prior cytoreduction, at high-risk for vascular complications, and with the presence of a *JAK2* V617F mutation.

In addition, we performed digital droplet PCR measurements with a sensitivity of 10^−3^ to quantify the *JAK2* V617F variant allele frequency (VAF) before and during pegIFN treatment [13]. In total, 70 quantifications were performed in 14 patients with the availability of DNA from peripheral blood granulocytes at three time points or more: the first sample within 1 year prior to pegIFN start and two or more samples at later time points during follow-up (each sample at least 1 year apart). Subsequently, a median number of 4.5 measurements (3–8) per patient was performed with a minimum follow-up of 2 years. The median follow-up time of *JAK2* V617F VAF measurements under pegIFN treatment was 5 years (2–8).

*JAK2* V617F quantification in 14 patients showed a median VAF of 30.5% (7.2–48.1) at pegIFN baseline, 8.3% after 4 years, and 18.1% after 8 years of pegIFN treatment (Fig. 1E). There were no statistically significant differences compared to baseline. Of note, 5/14 patients (36%) showed PMR at the last follow-up compared to baseline. All patients with PMR had a baseline VAF >10% (13.6–48.0), which decreased to 8.9% (median, range 0.99–19.4) at the last follow-up, and median time to ≥50% *JAK2* V617F VAF reduction was 4 years (2–5). Only one of the responders showed a VAF <1% (0.99%) at the last follow-up.

In 10/127 patients (8%), pegIFN-2b was stopped due to market withdrawal. Based on preliminary data showing that treatment-free remissions may be achieved in a subset of long-term pegIFN-treated patients, they were not immediately switched to an alternative drug as they had been treated for a median of 9.5 years (6–18) [14]. While 7/10 patients re-started pegIFN (or an alternative cytoreductive therapy) after a median treatment-free time of 7 months (3–15) due to an increased platelet count, 3/10 patients treated over 15–18 years maintained normal blood cell counts until data cut-off (1.5–4.5 years after discontinuation).

Interestingly, 4/7 patients with loss of complete hematologic response were *JAK2* V617F positive and all four patients showed an allele burden of >5% at the time of pegIFN discontinuation: 5.6%, 16.7%, 19.9%, and 41.7% (the remaining three patients were *CALR* mutated; VAF not available). In contrast, 2/3 of patients maintaining complete hematologic response had undetectable *JAK2* V617F (<0.1%), while 1/3 was at 0.1% at pegIFN stop in 2017. In this patient, *JAK2* V617F rose slowly to 0.54% after 4 years of follow-up without medication although PLT count remained normal. These three patients were not included in Fig. 1E since no DNA prior to pegIFN treatment (2001–2002) was available for quantification of *JAK2* V617F allelic burden. Bone marrow biopsies were performed in 2/3 of patients who maintained normal blood cell counts over 1.5 and 4.5 years after pegIFN discontinuation, respectively. It is instructive that, in both cases, histomorphologic remission of ET was demonstrated.

In summary, our data on 127 WHO-defined ET patients treated in routine clinical practice confirms the high efficacy of pegIFN in achieving a clinicohematologic response. Our study stands out for its unique sample size, a very long follow-up time of a subset of patients, and shows low rates of adverse events, vascular complications, and disease progressions. In the absence of randomized clinical trials and approval, our data support the use of pegIFN in ET patients. It is important to note that in individual cases it still remains difficult to distinguish ET from prefibrotic/early primary MF (prePMF). Therefore, one cannot fully exclude the possibility that some patients in our study had prePMF.

Prospective studies assessing *JAK2* allele burden monitoring and defining thresholds for planned treatment discontinuation in PV patients treated with ropegIFN are currently ongoing, and the results will be instructive for the management of ET as well [15]. In contrast to PV, quantification of mutated *CALR* remains an additional challenge in ET treated with pegIFN.
